# Where Do Adolescents Eat Less-Healthy Foods? Correspondence Analysis and Logistic Regression Results from the UK National Diet and Nutrition Survey

**DOI:** 10.3390/nu12082235

**Published:** 2020-07-27

**Authors:** Luigi Palla, Andrew Chapman, Eric Beh, Gerda Pot, Eva Almiron-Roig

**Affiliations:** 1 Department of Medical Statistics, London School of Hygiene and Tropical Medicine, London WC1E 7HT, UK; andrewandevachapman@yahoo.co.uk; 2 School of Tropical Medicine and Global Health, Nagasaki University, Nagasaki 852-8521, Japan; 3 School of Mathematical and Physical Sciences, University of Newcastle, Callaghan NSW 2308, Australia; eric.beh@newcastle.edu.au; 4 Department Nutritional Sciences, Faculty of Life Sciences & Medicine, King’s College London, London WC2R 2LS, UK; gerda.pot@kcl.ac.uk; 5 Louis Bolk Institute, Nutrition and Health Team, 3981 AJ Bunnik, The Netherlands; 6 Centre for Nutrition Research, University of Navarra, 31009 Pamplona, Spain; ealmiron@unav.es; 7 MRC Elsie Widdowson Laboratory, Cambridge CB2 0SL, UK; 8 Navarra Institute for Health Research (IdiSNa), 31008 Pamplona, Spain

**Keywords:** obesity, eating context, nutrient-poor foods, nutritional surveillance, adolescents, survey data analysis, data mining, correspondence analysis, biplots

## Abstract

This study investigates the relationship between the consumption of foods and eating locations (home, school/work and others) in British adolescents, using data from the UK National Diet and Nutrition Survey Rolling Program (2008–2012 and 2013–2016). A cross-sectional analysis of 62,523 food diary entries from this nationally representative sample was carried out for foods contributing up to 80% total energy to the daily adolescent’s diet. Correspondence analysis (CA) was used to generate food–location relationship hypotheses followed by logistic regression (LR) to quantify the evidence in terms of odds ratios and formally test those hypotheses. The less-healthy foods that emerged from CA were chips, soft drinks, chocolate and meat pies. Adjusted odds ratios (99% CI) for consuming specific foods at a location “other” than home (H) or school/work (S) in the 2008–2012 survey sample were: for soft drinks, 2.8 (2.1 to 3.8) vs. H and 2.0 (1.4 to 2.8) vs. S; for chips, 2.8 (2.2 to 3.7) vs. H and 3.4 (2.1 to 5.5) vs. S; for chocolates, 2.6 (1.9 to 3.5) vs. H and 1.9 (1.2 to 2.9) vs. S; and for meat pies, 2.7 (1.5 to 5.1) vs. H and 1.3 (0.5 to 3.1) vs. S. These trends were confirmed in the 2013–2016 survey sample. Interactions between location and BMI were not significant in either sample. In conclusion, public health policies to discourage less-healthy food choices in locations away from home and school/work are warranted for adolescents, irrespective of their BMI.

## 1. Introduction

Obesity is a global public health problem, particularly in developed countries where the growing longevity of populations implies a steadily increasing burden of chronic diseases including obesity and its associated co-morbidities [1]. Beside raising health care costs, obesity also reduces the quality of life and impacts on economic activity.

Current obesity rates coincide with dietary population trends showing a high consumption of fat-rich, sugar-rich and nutrient-poor foods (typically categorized as “less-healthy” or “non-core” foods by nutrition professionals) to the detriment of nutrient-dense, low energy density options (“healthier” or “core” foods) [2,3]. In the UK, for example, 13.5% and 14.1% of boys and girls, respectively, (1.5 to 18 years) were overweight in 2017, of which 19.4% (boys) and 15.1% (girls) were obese. During the same period, all population groups, including adolescents, had a mean fruit and vegetable intake below the 5 a day recommendation [4]. Although over the last 10 years, the proportion of 11–18-year-old children consuming free sugars, sugar-sweetened beverages (SSBs) and trans-fatty acids has decreased slightly, it remains still high. On the other hand, intake of some desirable nutrients such as vitamin A and folate has decreased [4]. The consumption of SSBs, in particular, has been associated with an increased BMI, an increased obesity risk and an increased cardiometabolic risk in adolescents [5], making this group highly vulnerable, especially the most economically and socially deprived [6,7,8]. For example, adolescents living in less-affluent households and boroughs are more likely to be exposed to and eat take-away meals at home or with peers than children from higher SES groups [9,10], which may increase their risk of adult obesity [8].

To counter these trends, several countries have devised policies to combat the development of poor eating habits, especially amongst young people, as overweight and obesity in teenage years tends to become established during adulthood [11]. In Britain, where the prevalence of adult obesity is higher than the Organisation for Economic Cooperation and Development (OECD) average [12], some policies were drafted as early as 2009 [13] to promote a healthy lifestyle and discourage unhealthy habits associated with weight gain from an early age.

Although obesity has a demonstrated heritable component [14], investigations conducted to date still point at the environment as a major determinant [15] and likely primary focus of intervention policies, in particular related to diet and physical activity [16,17]. The environment comprises many elements, including all influences that can be regarded as the social, economic, psychological, physical, geographical and political context in which behaviour takes place and which need to be considered. In relation to adolescents, the food environment has now been established as an important determinant of dietary behaviour [18,19], and therefore modifying the food environment has been suggested as a strategy to facilitate healthier eating behaviours [20,21]. Specifically, eating out of home in children and adolescents has been linked with the consumption of nutrient-poor, energy-dense foods, including SSBs, cakes, take-away meals and crisps [7,22,23]. While home food can be a significant contributor to total dietary intake in adolescents, food is typically obtained from a wide range of environments, particularly take-away, fast food and education establishments [23]. For instance, up to one-fifth of children in the UK may eat take-away meals at home once per week or more [9]. A more recent analysis of British children aged 1.5–18 years concluded that the home and school eating environments are associated with better food choices, while other locations including outlets selling foods to eat on the go are associated with poorer food choices [18]. These results mimic the rest of Europe and Canada, where the home and school environments are associated with a higher consumption of desirable nutrients and a diet lower in energy density in adolescents [24,25].

Qualitative research conducted in 18–25 years olds in Australia showed that intervention strategies suggested by young people themselves to reduce the consumption of specific less-healthy foods (e.g., energy drinks) included several environmental changes such as policy targeting sales, change in packaging, price, and visibility, in addition to more research and education [26]. However, before such policy can be implemented, a more in depth understanding of adolescent’s eating behaviour across eating locations is needed. For instance, the impact of the school food environment may be different for the younger (up to 10 year olds) than older children (11–18 year olds) [18], and from other population groups. However, not all previous literature has analysed these groups separately [9,18,23]. In addition, the modulating role of BMI on food choices by location remains inconclusive, with some studies suggesting a role [27], while others do not [10,18]. Finally, previous research in other countries has relied on menu offers or food-purchasing data [28], and has looked only at specific foods and/or locations [9,19].

As a further contribution to characterising the eating context in children, this paper investigates the relationship between the consumption of healthier and less-healthy food and eating location in British adolescents, specifically the likelihood of consuming foods of public health concern in out-of-home environments. For this, data from the United Kingdom National Diet and Nutrition Survey Rolling Programme (NDNS-RP) 2008–2012 database [29] and the NDNS-RP 2013–2016 [30] were explored focusing on 11 to 18-year-old children. The aim of the study was, first, to explore the association between foods (especially less-healthy ones) and the location where they are eaten (by correspondence analysis). The second aim was to quantify the evidence that adolescents are more likely to eat such foods in certain locations than in others (by logistic regression). Additionally, the effect of other potential factors known to affect eating patterns in the young (e.g., age, sex, ethnicity, BMI, smoking, alcohol drinking, SES, and weekend/weekday) [9] was also considered and the possible effect modifications, especially of BMI, on consumption at different locations.

## 2. Materials and Methods

### 2.1. The NDNS-RP 2008–2012 Sample

Data for this analysis were collected as part of the NDNS-RP in the UK between 2008 and 2012 [29]. The NDNS-RP, funded by Public Health England and the UK Food Standards Agency, is registered with the ISRTCN registry under study ID ISRCTN17261407 and received ethical approval from the Oxfordshire Research Ethics Committee.

The NDNS-RP sample was drawn from the UK Postcode Address File, a list of all the addresses in the UK. The addresses come from small geographical areas based on postcode sectors, randomly selected from across the UK. A list of 27 addresses was then randomly selected from each postcode sector. In total, 21,573 addresses from 799 postcodes in the UK were randomly selected for the survey between April 2008 and March 2011. The randomly selected individuals were asked to complete a detailed diary of their food and drink consumption over four consecutive days including, for most individuals, at least one weekend day. An interview was conducted to collect background information on dietary habits, socio-demographic status, lifestyle and physical activity. The response rate for completion of the diary and interview was 56%, which is around the expected response rate for this type of surveys [31].

A total of 884 adolescents aged 11 to 18 years completed a 4 day estimated food and drink diary, which also included data on where they ate, with whom, whether they were seated at a table and whether the TV was on or off [29]. This sample also included a proportion of 1.7% (15/884) of adolescents, completing only 3 days of the diary. Strata based on age group, sex and geographical region were used in the NDNS-RP to calibrate proportions in the sample with the whole population [32]. The weighting system used by the NDNS-RP involved two steps designed to compensate for sampling selection probabilities and reduce bias resulting from differential non-response by age, sex and region.

The 2013–2016 additional cross-sectional sample included another 1090 adolescents, with a completion rate of 53% and followed the general above described survey sampling methodology.

### 2.2. Dietary Data

Interviewers visited participants in their home, wherein they placed an unweighted food diary to be completed over four consecutive days by survey members. Survey members were provided with written instructions and asked to record everything they ate and drank over the four days, both at home and outside. To ensure compliance and completeness of recording, follow-up checks were scheduled by the interviewer on the second or third day of recording either in person or over the telephone [33,34].

Food diary entries were analysed considering each individual food entry as the unit of analysis. The food data were disaggregated and coded by the NDNS team using DINO (Diet In Nutrients Out) software [35] to obtain a nutritional analysis of each food entry. Each recorded item was assigned a suitable food and portion code using food composition data from the Department of Health’s (DH) NDNS Nutrient Databank. Where standard portion sizes were recorded in the diary using pictures provided, portion sizes were assigned from the Food Standard Agency’s (FSA) portion size book [36]. For composite items which can be split into their component parts, for example sandwiches, each individual component was separately coded. For validation of estimations of energy intake from the self-reported dietary records of food and drinks consumed, the NDNS-RP included a doubly labelled water (DLW) substudy of participants aged four years and over [37].

For the purpose of this study, all analyses were conducted purely on eating location instances, ignoring portion sizes (and consequently calories) and eating occasions (and consequently eating times), and concentrating on whether a food was consumed at a location or not. This likely reduced, but did not remove, the problem of underreporting food consumption in dietary surveys [37].

### 2.3. Classification of Food Groups and Locations

The NDNS database classifies the foods consumed and recodes them into 59 main food groups [38], many of which contribute very little to adolescents’ diets. In order to focus on the major foods for this age group, the contributions to total calorie intake of NDNS food groups were ranked for the adolescent subsample. Analyses have focused on the food groups which contribute the top 80% of calories (25 food groups), referred to here as P80. The 25th food group (least contributor) contributes less than 1.5% of the total calories in the UK teenagers’ diet in this sample. The same food groups were found to account for up to 80% of calories consumed by adolescents in the 2013–2014 sample.

The P80 food groups were subsequently classified as healthier, less healthy and neutral based on an adaptation of the UK Food Standards Agency-Ofcom (FSA-Ofcom model) nutrient profiling system [39,40], as described in Pechey et al. [41]. We chose this system as it has been shown to align well with health professionals’ healthiness perceptions of foods [42]. The FSA-Ofcom model is used in the UK to define less-healthy foods that cannot be advertised to children and calculates a score by adding points for greater presence of energy, saturated fat, total sugar, sodium and subtracting points from the score for presence of non-soluble fibre, protein, fruits, vegetables and nuts content [39]. An adaptation to this score was applied following previous work [3] and considering products’ nutritional characteristics, beginning with individual products so that a sharper delineation of food categories could be obtained vs. most previous work [41].

The P80 food groups were assigned the three categories used by Pechey et al. [41] as follows: healthier for scores below (−2), neutral between (−2) and (+4) inclusive, and less healthy for scores above (+4) (Table 1). Caution must be applied in interpreting this classification though as with any scores, some products can be classified counter-intuitively (e.g., chips and SSB as neutral).

Eating “location” was derived from the NDNS “where” codes as one of 7 categories: home, school, work, friends’/carers’/relatives’ homes, mobile, leisure or other. These categories were subsequently collapsed into three (home, school or work, other) based on results of the first exploratory analysis (see Results).

### 2.4. Statistical Analysis

The data available comprise a contingency table resulting from the cross-classification of many foods (reduced from 59 to 25) and locations (7). Despite the reduction in relevant foods, a total of 175 associations would have to be examined. These may encounter issues of lack of power and multiple testing. To circumvent that, a two-step analysis was conducted. In the first step (exploratory), data mining was applied by using multivariate techniques for hypothesis generation. This was followed (step 2) by a regression analysis to test in a separate sample the hypotheses generated in the first step regarding the association between foods and locations. For step 1, a contingency table was created from the food diary entries specifying the frequency of the consumption of each food group at each location. To identify potential associations between food groups and locations, simple correspondence analysis [43,44] was applied (without assuming a direction of the association in this exploratory step of the analysis), followed by visual inspection of correspondence analysis plots with confidence regions [45,46]. In step 2, the hypotheses thus generated were then taken forward and formally tested using logistic regression with food consumption as outcome and locations as exposures via Generalised Estimating Equations [47]. As more recent NDNS data became available for 4 additional years (2013–2016) since our first analyses, the hypotheses generated using the 2008–2012 survey sample were additionally tested by logistic regression in the 2013–2016 survey sample (n = 1090 adolescents) for confirmatory purposes. This was justified given that correspondence analysis plots derived for the 2013–2016 data indicated the same foods as for the 2008–2012 sample as potentially linked with specific locations (results not shown).

Analyses were conducted using SAS software version 9.4 and R package “CABOOTCRS” [45].

#### 2.4.1. Step 1: Correspondence Analysis (CA)

CA is a method for investigating the relationships between categorical variables represented in a two-dimensional contingency table. Within this paper’s context, it does this by analysing and visually depicting those food groups (and locations) that have a similar and differing “profile”, that is the relative frequency of the consumption of one food across different locations (or, symmetrically, the relative frequency of the consumption of different foods at one location) [43,44]. For example, if 71% of all foods consumed are eaten at home, but only 55% of all sweetened soft drinks are consumed at home, then sweetened soft drinks will have a location “profile” different from the average food profile. CA plots represent visually the chi-squared deviation (inertia) of food (and location) profiles from their respective average profile [43,44].

To plot these multidimensional deviations (inertia) reduced to the two most informative dimensions, biplots [48,49] were used where row profiles are normalised (rescaled) but column profiles are not (or vice versa). The horizontal axis of the biplot represents the direction along which the contingency table rows and columns show their largest deviation. The vertical axis represents the direction, perpendicular to the first, having the second-largest deviations. The percentage label for each axis is a measure of how much of the total variation (inertia) in the data has been displayed along that axis. The sum of the variation shown by the two axes is not 100%: the remaining variation would require displaying more dimensions, and so is lost when reducing to 2 dimensions. The origin in each plot represents the average profile of those points in the plot while the length of the vector from the origin to any profile point represents its deviation from the average profile. In biplots, the distance between row (food) and column (location) profile points and the direction in which they lie away from the origin is an indication of their association (greater association if points are located in similar directions away from the origin).

For this exploratory analysis (step 1), the whole survey design was not taken into account, because the statistical units analysed by CA were the food entries rather than the individuals sampled for the survey and the aim was to obtain a descriptive snapshot of the eating behaviour of British adolescents. However, since omitting weights have the potential to bias the analyses, sensitivity analyses were performed to verify that the interpretation of the CA plots did not change when assigning to each food entry either the respective individual’s weight or the individual weight divided by the total number of food entries for that individual (Figures S1–S9).

Confidence regions (CRs) based on the Ringrose bootstrap method [45] (with 95% confidence) were applied to identify whether foods were not significantly different from the average profile, i.e., whether the region contained the origin.

#### 2.4.2. Step 2: Logistic Regression with GEE

Logistic regression using Generalised Estimating Equations (GEEs) was applied to estimate odds ratios (ORs) of eating foods at different locations and test the null hypotheses that they were different from 1 for selected foods. GEEs provide unbiased estimates of ORs and valid estimates of standard errors even when the true correlation structure in the data is unknown [47,50,51,52]. To accommodate the potential variance inflating elements of the complex survey design (clusters and weights), a two-level hierarchical weighted analysis was conducted accounting for the correlation within survey design clusters at the first level (geographical areas that constitute the survey primary sampling units) and within individuals at the second level. The empirical standard errors were estimated assuming an exchangeable correlation matrix [51,53]. Both unadjusted odds ratios and fully adjusted (by potential confounders) odds ratios of eating foods at different locations were modelled to assess the stability of the estimates (although these are not directly comparable due to the non-collapsibility of odds ratios). Statistical significance for all regression analyses was set at 0.01.

## 3. Results

### 3.1. Study Sample

Data from 884 teenagers providing a total of 62,523 food entries were available for the 2008–12 survey. The mean number of food entries for an individual teenager was 71 (SD 8.5). The study sample consisted of 50.3% (445/884) boys and 49.7% (439/884) girls aged between 11 and 18 inclusive (Table 2). There were 20 (2.3%) missing values for the socio-economic classification and 32 (3.6%) for BMI, which appeared to be randomly distributed across age and sex. The descriptive characteristics of adolescents in the 2013-16 survey sample are presented in Supplementary Table S1.

The majority (70.8%) of food diary entries were recorded as eaten at home, with 14.2% recorded as eaten at school or work and the remainder (15%) at other locations (Table 3). The percentage of food entries recorded as eaten at work was very low (<2%).

Using a random process to split the diaries’ dataset for the subset of P80 foods (The entire survey sample was randomly divided into 2 parts. To do so, a random number from a uniform [0,1] distribution was produced for each food entry and such food entry was assigned to first half of sample if the number was <0.5 and to the second half of the sample if the number was ≥0.5.) resulted in a hypothesis-generating dataset of 20,567 food entries and a hypothesis-testing dataset of 20,455 food entries. The percentages of food entries at home, school/work were, respectively, 70.5% and 14.6% in the hypothesis-generating random sample and 71.2% and 14.1% in the hypothesis-testing sample.

### 3.2. Results from Correspondence Analysis (Step 1)

The initial CA plot (Figure S10) comprising all twenty-five P80 foods showed larger deviations from the average food profile for coated chicken, chips (french-fried potatoes) and sweetened soft drinks, which appeared in the graph in the same direction as leisure locations, while chocolates and meat pies appeared in the direction of mobile locations and other locations, and finally crisps, brown bread and biscuits appeared close enough to the school location. The home location attracted breakfast foods, pasta/rice, and vegetables. When adding confidence regions, the cluttered graph required creation of separate CA plots according to the healthiness classification presented in Table 1.

**Figure 1:**
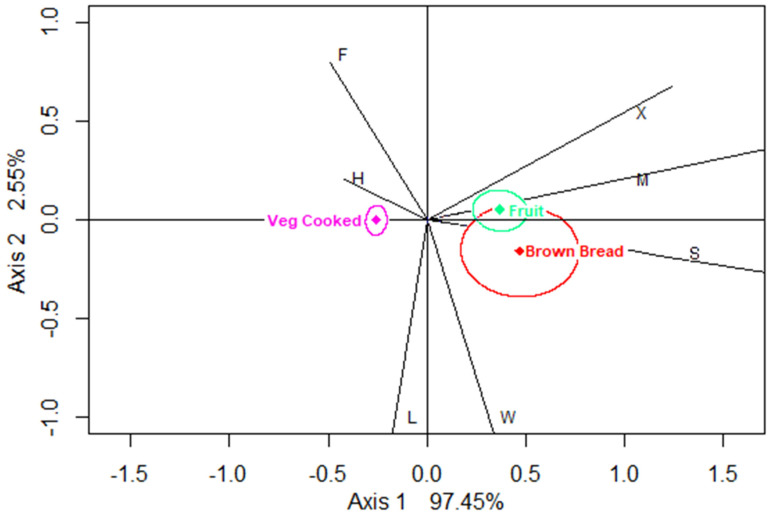
Biplot of locations showing confidence regions for healthier food groups (using CABOOTCRS). Legend: H—home, S—school, W—work, F—friends’/carers’ homes, L—leisure, M—mobile, and X—other.

The CA biplot for healthier food (which captured 100% inertia in the data) suggests that cooked vegetables tend to be associated with home, brown bread with school and fruit primarily with mobile locations (Figure 1). The three “healthier” foods have CRs not including the origin, meaning that across location, consumption patterns of cooked vegetables, brown bread and fruit are significantly different from the average healthier food (average across these three healthier foods).

**Figure 2:**
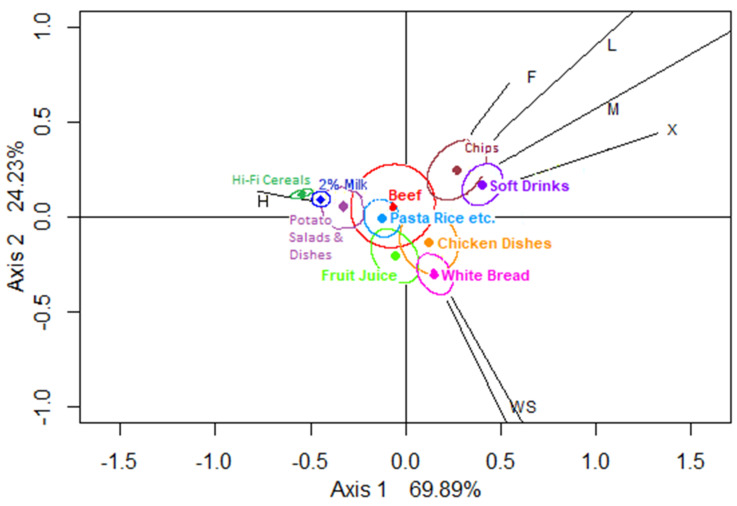
Biplot of locations showing confidence regions for neutral food groups (using CABOOTCRS). Legend: H—home, S—school, W—work, F—friends’/carers’ homes, L—leisure, M—mobile, and X—other.

The CA Biplot for neutral foods indicates that 94.1% of the inertia is captured in the plot (Figure 2), including 69.9% along the horizontal axis, where there is a contrast between home and all other eating locations, with leisure and mobile the most dissimilar to home. The vertical axis contrasts chips and soft drinks with white bread and fruit juice. The biplot for neutral foods suggests associations of school and work with chicken dishes, fruit juice and white bread and of leisure and mobile locations with chips and sweetened soft drinks. Beef and chicken dishes can be seen to have CRs which include the origin, whereas the CRs for chips and non-diet soft drinks do not include the origin, meaning the consumption locations of these two categories are significantly different from those of the average neutral food.

**Figure 3:**
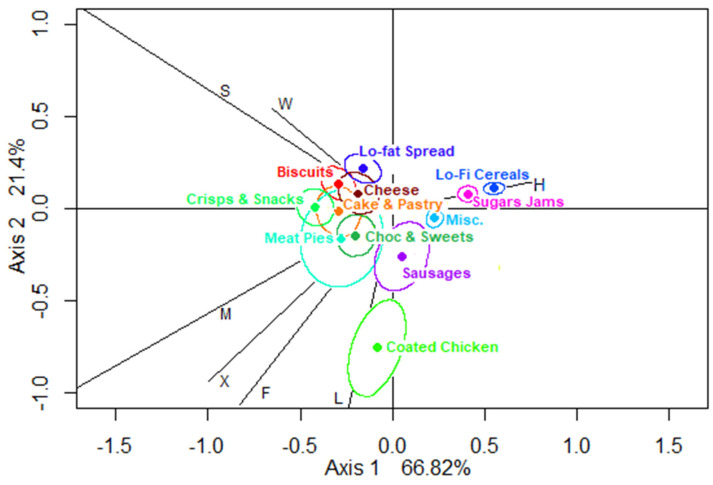
Biplot of locations showing confidence regions for less-healthy food groups (using CABOOTCRS). Legend: H—home, S—school, W—work, F—friends’/carers’ homes, L—leisure, M—mobile, and X—other.

The CA biplot for less-healthy foods (Figure 3) indicates that 88.22% of the variation in location profiles is captured in this plot, with 66.82% along the horizontal axis where home is contrasted with all other eating locations, and breakfast foods are contrasted with snack foods such as crisps, biscuits and cakes. The vertical axis again presents a contrast between fast foods and sandwich items.

The biplot for less-healthy foods suggests that (1) cheese, less-fat spreads and biscuits are associated with school (work); (2) crisps and cakes and sweet pastries are associated with non-home locations; (3) coated chicken is associated with leisure locations; (4) meat pies and chocolate appear associated with friends’ and carers’ homes, other and mobile locations. The chocolate CR appears entirely inside the CR for meat pies, suggesting that the consumption of chocolate and meat pies follows a very similar, almost indistinguishable pattern with regard to locations.

### 3.3. Overall Summary of Data Mining for Associations by Correspondence Analysis

In the CA biplots for neutral and less-healthy foods, the locations appear to have clustered in similar directions away from the origin in three main types (home, school/work, and other locations) which have been used to simplify the next analysis stage (hypothesis testing). The “other location” category includes leisure, mobile, other, friends’ and carers’ homes which were found in the same quadrant of either biplot. The latter collapse of location categories was not, however, suggested by the CA biplot of healthier foods, which comprised only a small number of food groups and of food entries, and therefore the healthier food category was not taken forward to the next stage of the analysis.

### 3.4. Results of Logistic Regression Analyses (Step 2)

After selecting meat pies, chocolate, chips, non-diet soft drinks, as they were lying away from the origin, in the same quadrant as other locations in the respective biplots at step 1, GEE logistic regression models were created to quantify the odds of consuming these specific neutral/less-healthy foods in other locations vs. the odds of their consumption at home or at school/work.

Results of the logistic regression analysis (Table 4) applied to the second (random) half of the sample showed that the average adolescent is more likely to consume sweetened soft drinks, chips, chocolate and meat pies at “other” locations rather than at home or at school/work. These results are statistically significant at the 1% level except for meat pies at other location versus school/work (*p* = 0.47).

The confirmatory analyses using the 2013–2016 NDNS-RP survey sample indicate the same main findings remain standing, although the odds ratio of other location vs. school/work is attenuated for chocolate and changed direction for meat pies (Table 5), compared to the 2008–2012 results.

Furthermore, the odds of having soft drinks (2.79 to 3.06) and chips (2.82 to 2.91) at other locations vs. home and of having soft drinks at other locations vs. school/work (2.02 to 2.50) increased (adjusted analyses in Table 4 and Table 5).

The adjusted model results (presented in Supplementary Table S2) further indicate that socio-economic status (SES) significantly affects the consumption of soft drinks and chips. In particular belonging to the third social class (intermediate occupations, SES3) results in 68% (99% CI: 6 to 264%) higher odds of consuming soft drinks compared to being in the baseline (modal) category SES2 (lower managerial, administrative and professional). Further, belonging to the fifth lower class (SES5, lower supervisory and technical) results in 47% (99% CI: 1 to 218%) higher odds of consuming soft drinks than for SES2. On the other hand, belonging to SES1 (higher managerial) results in 37% (99% CI: 20 to 86%) lower odds of consuming chips than SES2. However, there does not appear to be a linear trend in the effect of SES on the consumption of any food. White adolescents were 96% more likely to consume chocolate (99% CI 12% to 333%) than non-whites. Significant interactions between any confounding variables and location were not detected—in particular adolescents’ BMI was not modifying the odds ratio of consuming any of the selected foods in other locations compared to home and school. However, there was a significant interaction between smoking and ethnicity (the main effects of which were both very small and not significant) on the consumption of soft drinks. In particular, smoking non-white adolescents had 67% lower odds (99% CI: 20 to 86%) of consuming soft drinks than non-smoking whites. The comparison of crude to adjusted odds ratios showed remarkable stability of the estimates, which were virtually the same across models for soft drinks and chips.

The tendency of some social classes to significantly influence the consumption of chips and soft drinks and of ethnicity to influence the consumption of chocolate were found also in the 2013–2016 sample. The interaction between smoking and ethnicity, however, was not replicated. The model estimates were also generally stable across crude and adjusted analyses in the 2013–2016 sample.

## 4. Discussion

This study provides evidence that British adolescents are considerably more likely to consume specific nutrient-poor foods when they are away from home, school or work than when they are at these locations. Adolescents in this sample were nearly three times as likely to consume high-sugar and high-fat food when they were at other locations than when they were at home, in particular, sweetened soft drinks (279% increase), chocolate (256% increase), meat pies (273%) and chips (French fries) (282%). Similar results were found for the likelihood of eating such foods away from school or work (202% increase for soft drinks, 188% for chocolate and 342% for chips). Additionally, belonging to some lower SES groups was associated with a higher likelihood of consuming soft drinks and chips; and for the white ethnic group, with a higher likelihood of consuming chocolate.

Eating patterns for the consumption of healthier foods at home/school vs. at other locations were not so apparent in the CA biplot and therefore were not followed up in the subsequent regression analysis. This was partly due to only a few foods being classified as healthier and the fact that the comparisons in the biplot were made only within the small subset of healthier foods, with a consequent limited power of a follow-up regression analysis. However, the healthier food exploratory analysis did suggest that fruit, cooked vegetables and brown bread tend to associate with mobile, home and school locations, respectively, which may be worth exploring in future analyses.

### 4.1. Strengths and Weaknesses of the Study

This study used a nationally representative population of British adolescents for which food intake data were collected by means of an estimated food diary. This represents an advantage in regards to the use of proxy data for actual intake (e.g., purchase location or menu offerings), or the use of Food Frequency Questionnaires, which rely on memory, but, as with all self-reported data, it is prone to over or underestimative effects [55]. While misreporting generally applies to energy intake estimations, this analysis focused on the likelihood of consuming vs. not consuming specific foods at particular locations. Despite this still representing a limitation in that portion sizes of the same foods may vary both across individuals and brands [56,57,58], our approach has the advantage that underreporting effects are likely to be reduced, as portion size estimation errors were omitted altogether [55,59]. However, there may be residual bias due to the impact of recording a diary on eating behaviour or due to differential reporting of different foods (i.e., in terms of reporting vs. non-reporting rather than misreporting portion size).

The analysis included NDNS diary entries from the period 2008–2012. However, the ongoing nature of the NDNS rolling programme with its regular data releases provided a key opportunity for these analyses to be repeated for assessment of trends over time. A confirmatory analysis in the 2013–2016 sample confirmed the earlier findings, providing initial evidence for a secular trend in the consumption habits of adolescents at out-of-home locations over an 8 year period. Further confirmatory analyses in the most recent wave of the RP [4] are warranted.

The use of correspondence analysis with confidence regions facilitated mining the data in a preliminary broad exploration, which lead to a reduced risk of type I error (i.e., obtaining significant results by chance should a multiplicity of tests be carried out) [60,61]. Interpretation should be careful though in that these are relative results which do not convey information on the absolute number of eating occasions in various locations. Confirmation of the presence of fast food and take-away outlets by geocoding with actual eating location may have provided complementary evidence of the effect of the food environment [27]. However, presence of such outlets near the actual eating location does not guarantee adolescents are eating in such places. Equally, adolescents may be obtaining the food in these outlets and consuming it elsewhere [9].

Data on social facilitation (i.e., eating more in the presence of others) were not analysed. However, this may be an important factor given the effects of peer pressure on food choices in adolescents [26]. For instance, peer influence has been shown to be a major contribution to the consumption frequency of fast food in adolescents from deprived boroughs [10], and is also a contributing factor to the consumption of snacks high in solid fats and added sugars at friends’ homes [62]. On the other hand, the diets of adolescents are more difficult to be controlled by parents compared to those of younger children because, as children age, they develop more independent eating habits. In addition, parental support for healthier habits tends also to be stronger in families of higher SES [6,18].

The use of the FSA-Ofcom model to classify food as less healthy has recently been put into question based on its potential inconsistency to discriminate among foods with respect to their association with specific diet-related diseases [63]. One likely inconsistency in this system was the classification of chips and non-diet soft drinks in the neutral category and its comparison to other neutral rather than less-healthy foods in the CA exploratory analysis. However, this did not affect the final results estimating the odds of these foods being consumed at other locations vs. at home. Using this system, only three foods were classified as “healthier” in the present study, which may reflect the system not being originally designed to identify healthier foods [40]. However, the overall results strongly support findings from two parallel analyses in British children and adults using a different classification system [18,64], indicating that the discriminatory capacity of the adapted FSA-Ofcom model in the context of this study is probably consistent with that of other systems currently in use.

### 4.2. Comparison with Previous Studies

To our knowledge, this is the first study to specifically quantify the impact of the food environment on the self-reported consumption of foods of concern in a nationally representative sample of the teenage population in the UK. Past studies with British adolescents have been mostly descriptive and included adults, younger children or smaller samples and focused on frequency of consumption in specific locations only [7,9,23,65]. Studies in other countries did not use direct food intake data [28] or examine consumption in specific locations only [19,66,67] or from specific food groups [22,23]. Only two studies to date in British adolescents have used direct food intake data to explore eating context influences on diet [18,23], and our results fully support their findings. Ziaudeen et al. [18] reported that food outlets, leisure places, and “on-the-go” locations were the out-of-home food environments associated with the highest proportion of energy from noncore foods, which include sweetened soft drinks, chips, chocolates and pastries. Similarly, Tyrrell et al. [23] reported that amongst 16–22 year olds, the main sources of energy, fat and sugar were foods purchased at convenience and specialist shops, retail bakers, vending machines and take-away establishments. It can be concluded then that adolescents’ food choices are strongly influenced by the food environment, with the purchase and consumption of high-sugar and saturated-fat foods associated with away from home and from school locations, while the consumption of desirable nutrients and lower dietary energy density is linked with eating at home and school [9,18,19,22,23,67].

Our results also confirm previous reports on the clustering of less-healthy eating behaviours at certain locations [10]. For example, the meat pie group includes sausage rolls, Cornish pasties, and meat pastries, which are all convenient foods to carry and eat at any location. The same applies to chocolate confectionery, all of which are available on every high street at a price point below £1 alongside soft drinks, providing a favourable environment for adolescents to consume such foods. In support of these observations, Patterson et al. [10] reported that teenagers in a deprived London borough spent a median of £2 and an upper quartile of £3 when buying fast food. Sausage rolls and meat pastries are also available from school lunch counters, but chocolate at school will not be at a promotional price if available at all. This may explain why the odds of eating meat pies at school/work locations are comparable to other locations, whereas for chocolate they are not.

In the present study, adolescents from families with a lower socio-economic class had higher odds of consuming chips and sweetened soft drinks independently of location. This is consistent with numerous previous studies in adults and children showing a link between fast food outlets, diet quality and the social environment, as take-away and fast food outlets tend to be relatively more present in more deprived areas [9,10,68,69]. In general, lower socio-economic classes tend to purchase a greater proportion of their energy from less-healthy items [41], consume more take-away food and live in areas with a higher proportion of fast food outlets [8,9], which is also consistent with our findings.

### 4.3. Interpretation of the Findings and Implications for Public Health Policy

Overall, our results agree with recent and previous work showing an association between the eating environment and food choices in adults, school-aged children and adolescents alike [9,18,23,64] but further quantify this association for foods commonly consumed by adolescents. Access to healthy food as part of school initiatives is probably an important factor to improve dietary choices, as shown in an analysis of children aged 1.5 to 18 using a related sample [18]. On the other hand, the lack of affordable healthy food in out-of-home and school environments may act as a prompt for less favourable eating choices in adolescents. As seen for younger children [18], this study still highlights the home environment as an important target for intervention given the high proportion of food entries recorded in this location. At the same time, it provides clear, strong evidence of the association between the food environment and the consumption of popular high energy density, nutrient-poor foods in a nationally representative sample of adolescents, warranting the need to improve food choices for this age group in environments outside the home and school.

The present results are particularly relevant for policy makers in the context of the current Childhood Obesity Strategy [70], as they allow gaining a broader understanding of the potential impact of the food environment in young people. In particular, the role of food cost, advertising and choice architecture (i.e., altering the environment to make healthier choices easier) [71] need to be considered. Future research should explore these and other incentives to make more healthy choices available and attractive to teenagers when they are “grazing” for food away from adult supervision.

## Figures and Tables

**Table 1:** Top 25 “P80” food groups sorted by increasing cumulative % of total calories consumed by adolescents in the NDNS-RP (2008–2012) and by FSA-adapted nutrient profile score [41].

	Calories Cumul %	Score	Healthiness Category		
			Healthier < −2	Neutral	Less Healthy > 4
Pasta and rice and other cereals	10.05	2.0		N	
White Bread	18.49	1.7		N	
Chips and potatoes	24.54	−0.3		N	
Soft drinks, not diet	29.86	2.2		N	
Biscuits	34.00	18.9			L
Crisps, savoury snacks	37.84	12.3			L
Chocolate (incl. confectionary)	41.50	25.2			L
Buns, cakes, sweet pastries, fruit pies	44.99	17.1			L
Chicken dishes and turkey	48.36	−0.5		N	
Miscellaneous unclassified foods	51.22	9.4			L
Cheese	53.88	22.0			L
Semi-skimmed milk	56.47	−0.5		N	
Vegetables (not raw)	58.93	−6.3	H		
Low-fibre breakfast cereals	61.20	11.8			L
Sausages	63.36	14.6			L
Coated chicken and turkey manuf.	65.37	5.6			L
Potatoes other, potato salads and dishes	67.24	−1.7		N	
Beef, veal and dishes	69.10	0.5		N	
Fruit	70.93	−3.3	H		
High fibre breakfast cereals	72.76	2.1		N	
Fruit juice	74.53	1.5		N	
Spreads, less fat	76.27	22.9			L
Meat pastries, rolls and pies (“meat pies”)	77.92	15.1			L
Brown Bread granary and wheat germ	79.42	−3.0	H		
Sugars, preserves and sweet spreads	80.91	15.1			L

**Table 2:** Participant characteristics. Data are from the National Diet and Nutrition Survey Rolling Programme Years 1–4 (2008–2012) for all respondents aged 11 to 18 years [29].

		N	%	% (Weighted Sample)
Age (years)	11–15	543	61.4	60.8
	16–18	341	38.6	39.2
Sex	Male	445	50.3	51.3
	Female	439	49.7	48.7
Ethnicity (%)	White	778	88	86.2
	Non-white	106	12	13.8
Occupational group (SES) *	1. Higher managerial, administrative and professional occupations	126	14.3	14.6
	2. Lower managerial, administrative and professional occupations	236	26.7	24.6
	3. Intermediate occupations	73	8.3	7.4
	4. Small employers and own account workers	94	10.6	11.2
	5. Lower supervisory and technical occupations	90	10.2	9.7
	6. Semi-routine occupations	125	14.1	14.6
	7. Routine occupations	91	10.3	11.5
	8. Never worked and long-term unemployed	29	3.3	4.3
	Missing	20	2.3	2.2
BMI (%)	Normal weight	553	62.6	62.7
	Overweight	124	14.0	14.5
	Obese	175	19.8	19.8
	Missing	32	3.6	3
Drinking (%)	Yes **	132	14.9	13.8
	No (once or twice a months or less)	752	85.1	86.2
Smoking (%)	Yes ***	91	10.3	10.4
	No	793	89.7	89.6

* nssec8 social and economic status classification from the Office for National Statistics [54]. ** Collapsed from the following original categories: almost every day, twice a week, once a week, and once a fortnight. *** Collapsed Smoking (category: current smoker) and Smoking Frequency (category: smoke cigarettes once a week or more often) variables.

**Table 3:** Distribution of total adolescents’ food diary entries by eating location in the 2008–2012 NDNS sample [29].

Location	Frequency	% of Total	Cumulative %
Home (all home locations)	44,271	70.8	
Home living room	15,552	24.9	24.9
Home kitchen	12,558	20.1	45.0
Home dining room	6207	9.9	54.9
Home bedroom	4100	6.6	61.5
Home not given	2703	4.3	65.8
Home other	2617	4.2	70.0
Home garden	534	0.9	70.8
School	7683	12.3	83.1
Leisure clubs, cafes	3190	5.1	88.2
Friends’/Carers’/Relatives’ homes	2769	4.4	92.6
Other locations	2161	3.5	96.1
Mobile: car, bus, train, etc.	1295	2.1	98.2
Work	1154	1.9	100
All locations	62,523	100	

**Table 4:** Odds ratio estimates of eating sweetened soft drinks, chips, chocolate and meat pies in other locations versus at home or versus at school/work, unadjusted and adjusted by age (continuous), sex, day of the week (weekdays vs. weekend), socio-economic status (eight categories), BMI (continuous), ethnic group (white or non-white), smoking status (smoker or non-smoker) and alcohol status (drinker or non-drinker).

	Unadjusted Odds Ratio				Adjusted Odds Ratio			
	Other Location vs. Home		Other Location vs. School/Work		Other Location vs. Home		Other Location vs. School/Work	
Food	OR	99% CI *p*-Value	OR	99% CI *p*-Value	OR	99% CI *p*-Value	OR	99% CI *p*-Value
Sweetened soft drinks (*N* = 1678)	2.78	(2.07, 3.73) *p* < 0.0001	2.09	(1.50, 2.93) *p* < 0.0001	2.79	(2.08, 3.75) *p* < 0.0001	2.02	(1.43, 2.84) *p* < 0.0001
Chips (*N* = 664)	2.81	(2.17, 3.63) *p* < 0.0001	3.42	(2.16, 5.40) *p* < 0.0001	2.82	(2.17, 3.66) *p* < 0.0001	3.42	(2.13, 5.50) *p* < 0.0001
Chocolate (*N* = 574)	2.49	(1.81, 3.42) *p* < 0.0001	1.72	(1.14, 2.60) *p* = 0.0007	2.56	(1.85, 3.51) *p* < 0.0001	1.88	(1.22, 2.91) *p* = 0.0002
Meat pies (*N* = 124)	2.61	(1.42, 4.81) *p* < 0.0001	1.22	(0.55, 2.71) *p* = 0.53	2.73	(1.48, 5.06) *p* < 0.0001	1.28	(0.53, 3.07) *p* = 0.47

The number of food entries per food out of total of 19,419 for complete case analysis are shown in brackets (N). Data are from the 2008–2012 NDNS-RP survey sample (*n* = 884 adolescents) [29].

**Table 5:** Confirmatory odds ratio estimates based on the 2013–2016 NDNS-RP survey sample (*n* = 1090 adolescents) [30] of eating sweetened soft drinks, chips, chocolate and meat pies in other locations versus at home or versus at school/work, unadjusted ORs and ORs fully adjusted by age (continuous), sex, day of the week (weekdays vs. weekend), socio-economic status (eight categories), BMI (continuous), ethnic group (white or non-white), smoking status (smoker or non-smoker) and alcohol status (drinker or non-drinker).

	Unadjusted Odds Ratio				Adjusted Odds Ratio			
	Other Location vs. Home		Other Location vs. School/Work		Other Location vs. Home		Other Location vs. School/Work	
Food	OR	99% CI *p*-Value	OR	99% CI *p*-Value	OR	99% CI *p*-Value	OR	99% CI *p*-Value
Sweetened soft drinks (*N* = 2999)	3.08	(2.56, 3.70) *p* < 0.0001	2.59	(1.96, 3.42) *p* < 0.0001	3.06	(2.53, 3.71) *p* < 0.0001	2.50	(1.84, 3.39) *p* < 0.0001
Chips (*N* = 1546)	2.92	(2.39, 3.55) *p* < 0.0001	2.94	(2.10, 4.12) *p* < 0.0001	2.91	(2.36, 3.57) *p* < 0.0001	2.75	(1.91, 3.95) *p* < 0.0001
Chocolate (*N* = 1440)	2.38	(1.84, 3.07) *p* < 0.0001	1.30	(0.94, 1.81) *p* = 0.0007	2.35	(1.82, 3.03) *p* < 0.0001	1.31	(0.93, 1.83) *p* = 0.04
Meat pies (*N* = 306)	1.96	(1.23, 3.10) *p* = 0.0002	0.76	(0.40, 1.45) *p* = 0.28	1.89	(1.19, 3.01) *p* = 0.0004	0.74	(0.39, 1.39) *p* = 0.21

The number of food entries (including all foods) out of total of 80,926 for complete case analysis are shown in brackets (N).

## Supplementary Materials

The following are available online at https://www.mdpi.com/2072-6643/12/8/2235/s1. Table S1: Participant Characteristics 2013–2016. Table S2: Estimated Odds Ratios (with 99% CI and *p*-values) for All Potential Confounders in the Adjusted Logistic Regression of Locations (Exposure) on 4 Food Outcomes in Table 4 (for the 2008–2012 Survey Sample) and Table 5 (2013–2016 Survey Sample). Figure S1: Correspondence Analysis Plot for Healthier Foods, without NDNS Weights. Figure S2: Correspondence Analysis Plot for Healthier Foods, with Individual NDNS Weights. Figure S3: Correspondence Analysis Plot for Healthier Foods, with Adjusted NDNS Weights. Figure S4: Correspondence Analysis Plot for Neutral Foods, without NDNS Weights. Figure S5: Correspondence Analysis Plot for Neutral Foods, with Individual NDNS Weights. Figure S6: Correspondence Analysis Plot for Neutral Foods, with Adjusted NDNS Weights. Figure S7: Correspondence Analysis Plot for Less-Healthy Foods, without NDNS Weights. Figure S8: Correspondence Analysis Plot for Less-Healthy Foods, with Individual NDNS Weights. Figure S9: Correspondence Analysis Plot for Less-Healthy Foods, with Adjusted NDNS Weights. Figure S10: Correspondence Analysis Including All 25 Main (P80) Food Groups for the 2008–2012 Survey Sample.
