# Supplementary material for: Where Do Adolescents Eat Less-Healthy Foods? Correspondence Analysis and Logistic Regression Results from the UK National Diet and Nutrition Survey
Source: Nutrients. 2020 Jul 27;12(8):2235. doi: 10.3390/nu12082235 (PMC7468703; doi:10.3390/nu12082235)

SUPPLEMENTARY MATERIAL

		N	%	% (weighted sample)
Age (years)	11-15	650	59.6	61.2
	16-18	440	40.4	38.8
Sex	Male	538	49.4	51.4
	Female	552	50.6	48.6
Ethnicity(%)	White	939	86.1	80.1
	Non-white	149	13.7	19.7
Occupational group (SES)*	1. Higher managerial, administrative and professional occupations	174	16.0	16.7
	2. Lower managerial, administrative and professional occupations	261	23.9	22.9
	3. Intermediate occupations	113	10.4	9.9
	4. Small employers and own account workers	150	13.8	15.0
	5. Lower supervisory and technical occupations	89	8.2	8.0
	6. Semi-routine occupations	142	13.0	12.1
	7. Routine occupations	114	10.5	10.0
	8. Never worked and long-term unemployed	32	2.9	3.9
	Missing	15	1.4	1.6
BMI (%)	Normal-weight	687	63.0	64.2
	Over-weight	130	11.9	11.2
	Obese	234	21.5	20.6
	Missing	39	3.6	3.9
Drinking (%)	Yes **	114	10.5	9.4
	No (Once or twice a months or less)	976	89.5	90.6
Smoking (%)	Yes ***	72	6.6	6.2
	No	1018	93.4	93.8

Table ST1: Participant characteristics 2013-2016. Data are from the National Diet and Nutrition Survey Rolling Programme Years 5-8 (2013-2016) for all respondents aged 11 to 18 years.

(A)2008-12 Sample	Sweetened Soft Drinks		Chips		Chocolate		Meat Pies	
**Confounder (after adjustment by locations)**	**OR**	**99% CI p-value**	**OR**	**99% CI p-value**	**OR**	**99% CI p-value**	**OR**	**99% CI p-value**
**Sex (Female)**	0.84	(0.66, 1.06) p=0.05	1.05	(0.83, 1.32) p=0.61	1.12	(0.83, 1.51) p=0.32	1.06	(0.61, 1.86) p=0.78
**Age**	1.04	(0.98, 1.11) p=0.09	0.99	(0.94, 1.06) p=0.82	0.96	(0.89, 1.04) p=0.19	1.13	(0.96, 1.33) **p<0.0001**
**SES (1)**	0.98	(0.64, 1.51) p=0.90	0.63	(0.43, 0.93) **p=0.002**	0.87	(0.89, 1.04) p=0.19	1.87	(0.75, 4.65) p=0.08
**SES (3)**	1.67	(1.06, 2.64) **p=0.004**	0.92	(0.60, 1.41) p=0.61	0.52	(0.26, 1.04) p=0.02	1.76	(0.59, 5.29) p=0.21
**SES (4)**	1.04	(0.64, 1.72) p=0.82	1.07	(0.72, 1.59) p=0.68	0.88	(0.55, 1.42) p=0.50	2.22	(0.84, 5.87) p=0.04
**SES (5)**	1.48	(1.01, 2.19) **p=0.009**	0.89	(0.54, 1.47) p=0.59	1.10	(0.63, 1.93) p=0.65	1.96	(0.58, 6.64) p=0.16
**SES (6)**	1.33	(0.92, 1.92) p=0.05	1.14	(0.77, 1.67) p=0.39	0.83	(0.51, 1.34) p=0.31	1.78	(0.76, 4.15) p=0.08
**SES (7)**	1.27	(0.83, 1.94) p=0.14	1.26	(0.87, 1.84) p=0.11	0.99	(0.60, 1.64) P=0.95	2.22	(0.86, 5.69) p=0.03
**SES (8)**	1.58	(0.93, 2.68) p=0.03	0.93	(0.41, 2.12) p=0.82	0.69	(0.26, 1.81) P=0.32	0.60	(0.04, 9.39) p=0.64
**Smoker**	0.91	(0.60, 1.38) p=0.56	1.17	(0.70, 1.94) p=0.43	1.03	(0.51, 2.09) P=0.92	1.52	(0.54, 4.25) p=0.29
**Drinker**	1.11	(0.76, 1.60) p=0.48	0.78	(0.51, 1.18) p=0.12	0.99	(0.59, 1.67) P=0.96	0.92	(0.39, 2.17) p=0.80
**Non-White**	0.99	(0.72, 1.38) p=0.99	0.88	(0.60, 1.29) p=0.39	0.51	(0.30, 0.89) **P=0.002**	0.71	(0.27, 1.84) p=0.35
**BMI**	1.00	(0.97, 1.03) p=0.80	1.02	(0.99, 1.04) p=0.12	0.97	(0.93, 1.01) P=0.03	0.99	(0.93, 1.07) p=0.82
**Weekend**	1.09	(0.89, 1.32) p=0.28	1.03	(0.81, 1.31) p=0.72	0.88	(0.67, 1.15) P=0.22	0.83	(0.44, 1.57) p=0.45
**Non-White&Smoker**	0.34	(0.14, 0.80) **p=0.001**	-	-	-	-	-	-

(B) 2013-16 Sample	Sweetened Soft Drinks		Chips		Chocolate		Meat Pies	
**Confounder (after adjustment by locations)**	**OR**	**99% CI p-value**	**OR**	**99% CI p-value**	**OR**	**99% CI p-value**	**OR**	**99% CI p-value**
**Sex (Female)**	1.04	(0.82, 1.33) p=0.65	0.92	(0.76, 1.11) p=0.26	1.01	(0.80, 1.29) p=0.87	0.71	(0.47, 1.09) p=0.04
**Age**	1.04	(0.98, 1.10) p=0.11	1.02	(0.97, 1.07) p=0.31	0.95	(0.90, 1.01) p=0.02	1.07	(0.97, 1.19) p=0.07
**SES (1)**	0.68	(0.47, 0.99) p=0.007	0.73	(0.53, 1.01) p=0.01	1.14	(0.77, 1.70) p=0.39	0.86	(0.41, 1.79) p=0.59
**SES (3)**	1.17	(0.81, 1.70) p=0.27	1.09	(0.78, 1.51) p=0.52	1.13	(0.73, 1.75) p=0.49	1.37	(0.66, 2.87) p=0.27
**SES (4)**	1.16	(0.75, 1.79) p=0.38	1.09	(0.79, 1.50) p=0.51	1.23	(0.84, 1.80) p=0.16	1.64	(0.83, 3.25) p=0.06
**SES (5)**	1.22	(0.77, 1.92) p=0.27	1.41	(1.03, 1.93) **p=0.005**	1.16	(0.73, 1.84) p=0.40	1.72	(0.82, 3.59) p=0.06
**SES (6)**	1.02	(0.67, 1.57) p=0.89	1.19	(0.86, 1.66) p=0.17	0.90	(0.60, 1.32) p=0.47	1.32	(0.66, 2.65) p=0.31
**SES (7)**	1.25	(0.81, 1.91) p=0.19	1.11	(0.82, 1.50) p=0.37	1.05	(0.70, 1.59) p=0.74	1.65	(0.74, 3.69) p=0.11
**SES (8)**	0.97	(0.48, 1.97) p=0.92	1.76	(1.22, 2.53) p<0.0001	1.24	(0.62, 2.48) p=0.43	0.64	(0.11, 3.68) p=0.51
**Smoker**	1.36	(0.79, 2.33)	1.49	(1.07, 2.07)	1.10	(0.67, 1.81)	0.89	(0.39, 2.05)

		p=0.15		**p=0.002**		p=0.62		p=0.73
**Drinker**	1.01	(0.66, 1.56)<br>p=0.94	1.05	(0.77, 1.43)<br>p=0.70	1.03	(0.70, 1.50)<br>p=0.86	1.02	(0.52, 2.01)<br>p=0.93
**Non-White**	0.99	(0.73, 1.33)<br>p=0.91	0.95	(0.70, 1.27)<br>p=0.63	0.64	(0.45, 0.92)<br>**P=0.001**	0.29	(0.12, 0.71)<br>**p=0.0004**
**BMI**	0.99	(0.96, 1.02)<br>p=0.41	0.99	(0.97, 1.01)<br>p=0.17	0.99	(0.79, 1.37)<br>P=0.31	0.98	(0.93, 1.03)<br>p=0.25
**Weekend**	1.13	(0.96, 1.33)<br>p=0.05	1.17	(1.00, 1.38)<br>p=0.01	1.04	(0.79, 1.37)<br>P=0.71	1.05	(0.70, 1.57)<br>p=0.77
**Non-White&Smoker (interaction)**	2.39	(0.74, 7.69)<br>p=0.06	-	-	-	-	-	-

Table ST2: Estimated odds ratios (with 99% CI and p-values) for all potential confounders in the adjusted logistic regression of locations (exposure) on 4 food outcomes in Table 4 (for the 2008-12 survey sample) and Table 5 (2013-16 survey sample).

Reference categories: male; SES=2; non-smoker; non-drinker; white; weekday.

For the sweetened soft drink outcome, interaction between ethnicity and smoking was also estimated as it appeared significant.

Figure S1: Correspondence analysis plot for Healthier Foods, without NDNS weights.

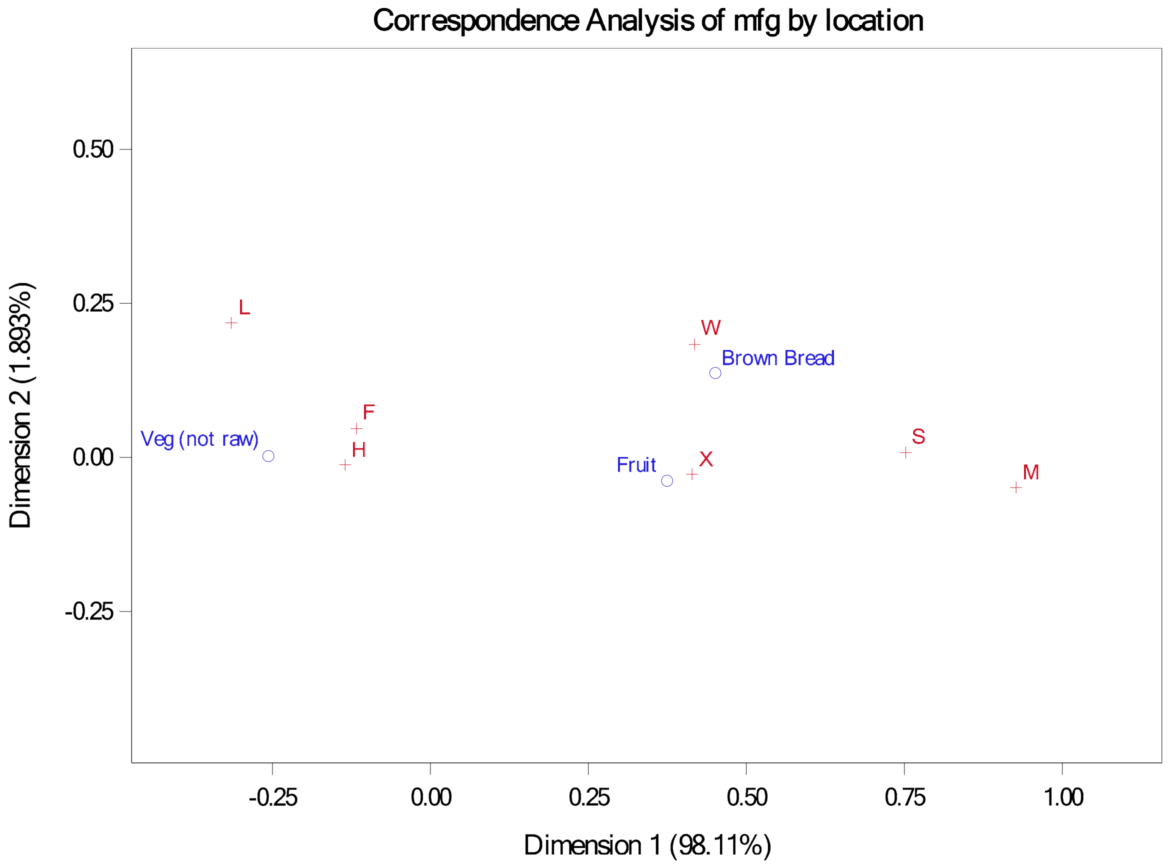

Figure S2:Correspondence analysis plot for Healthier Foods,with individual NDNS weights.

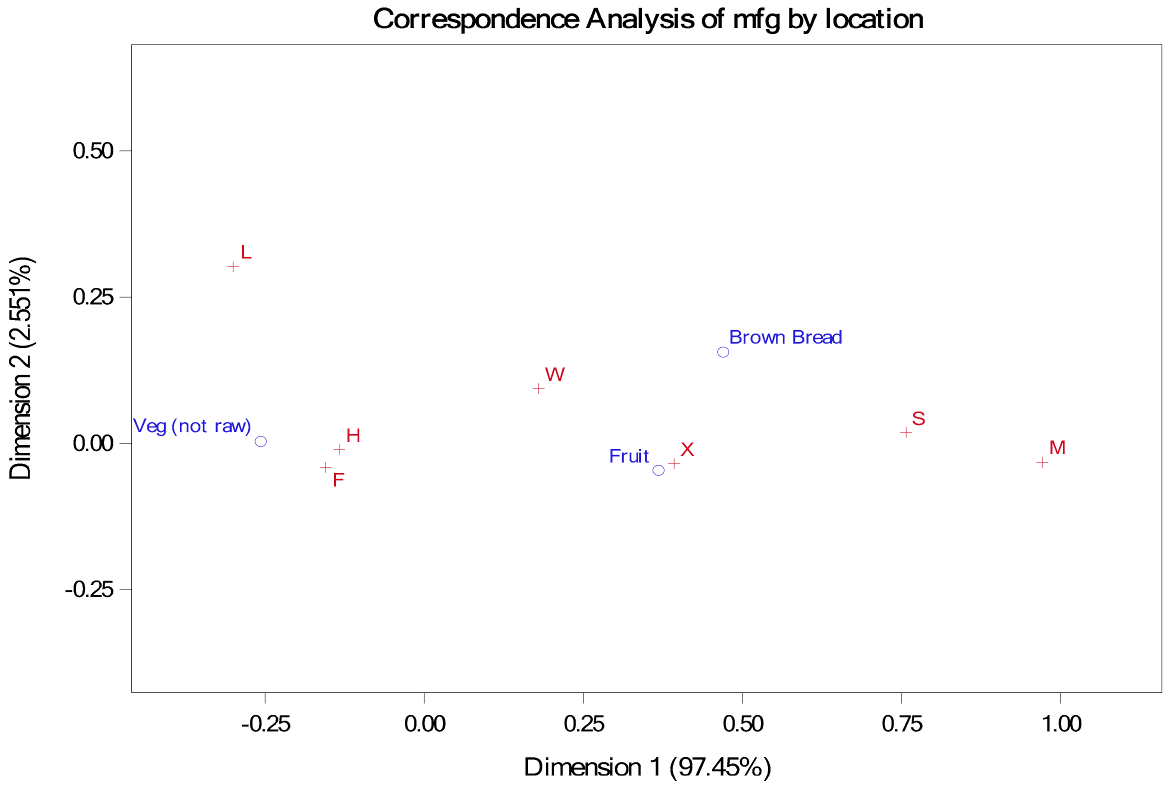

Figure S3: Correspondence analysis plot for Healthier Foods, with adjusted NDNS weights (individual's weight divided by number of food entry for that individual)

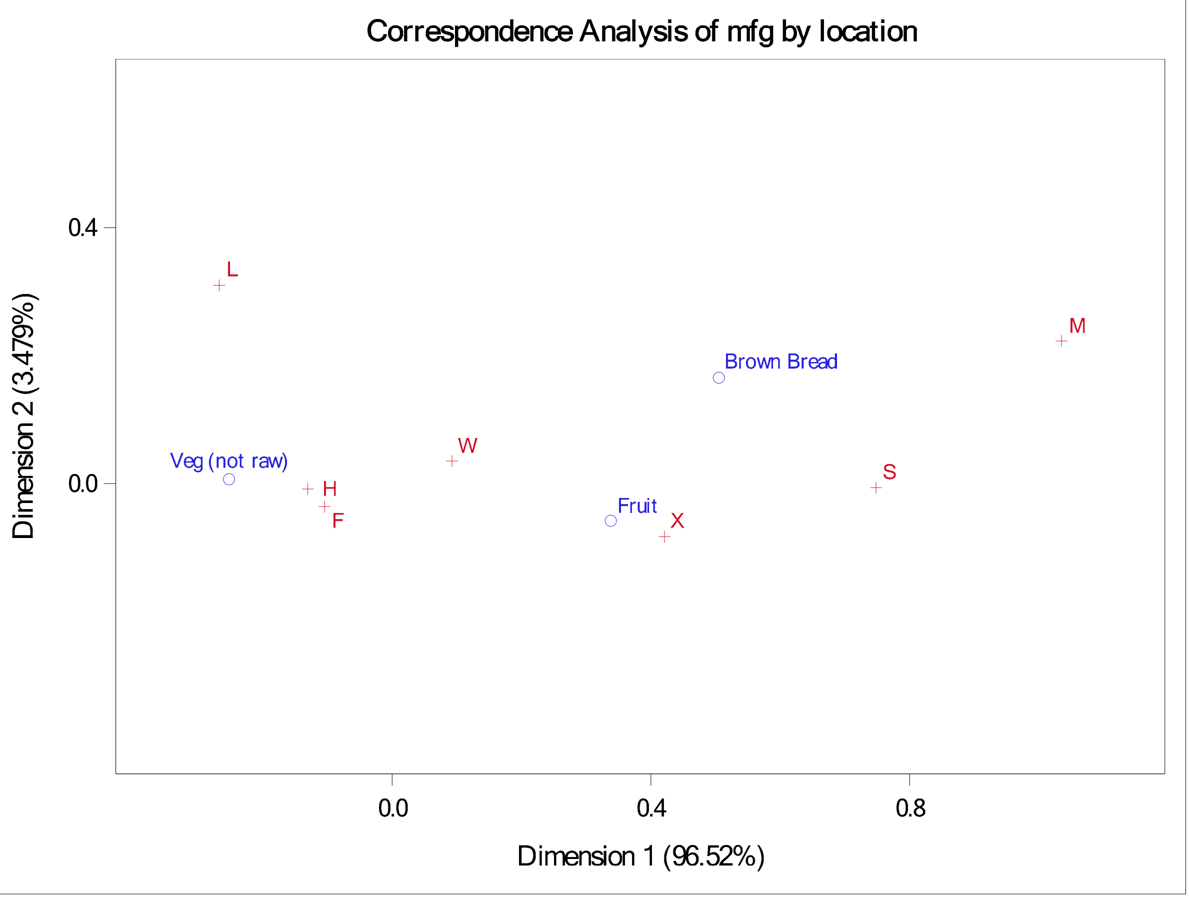

Figure S4: Correspondence analysis plot for Neutral Foods, without NDNS weights.

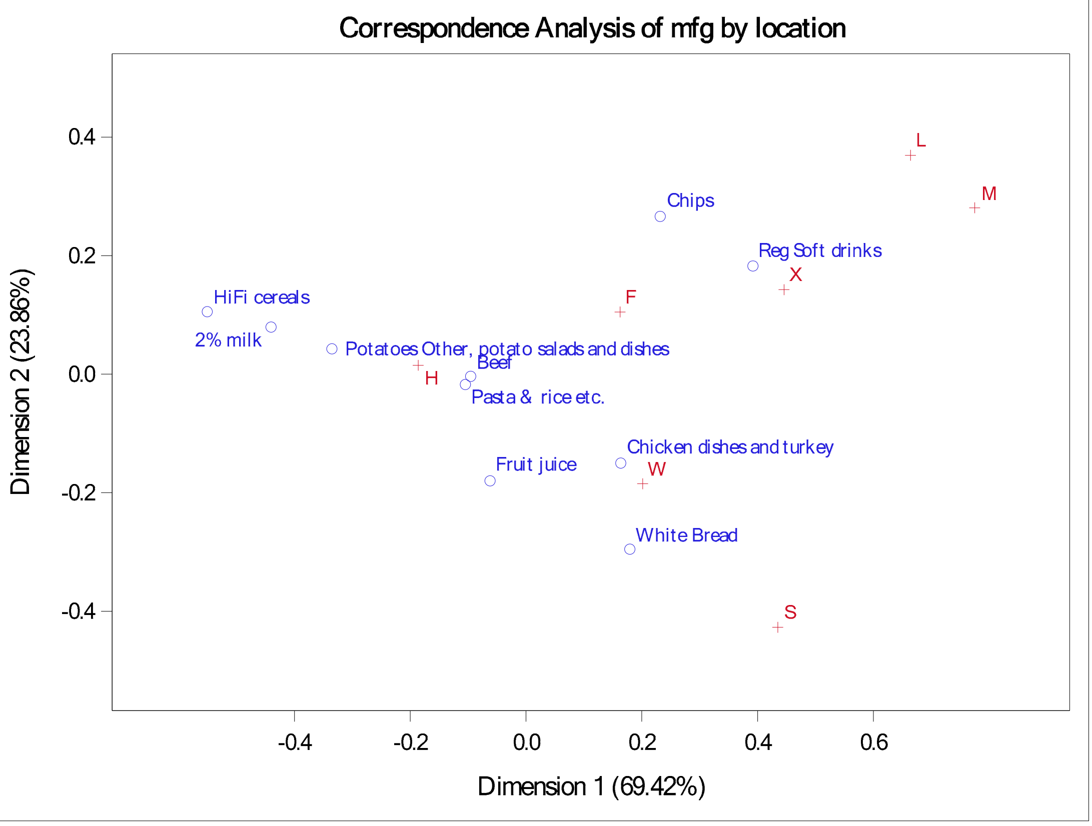

Figure S5: Correspondence analysis plot for Neutral Foods, with individual NDNS weights.

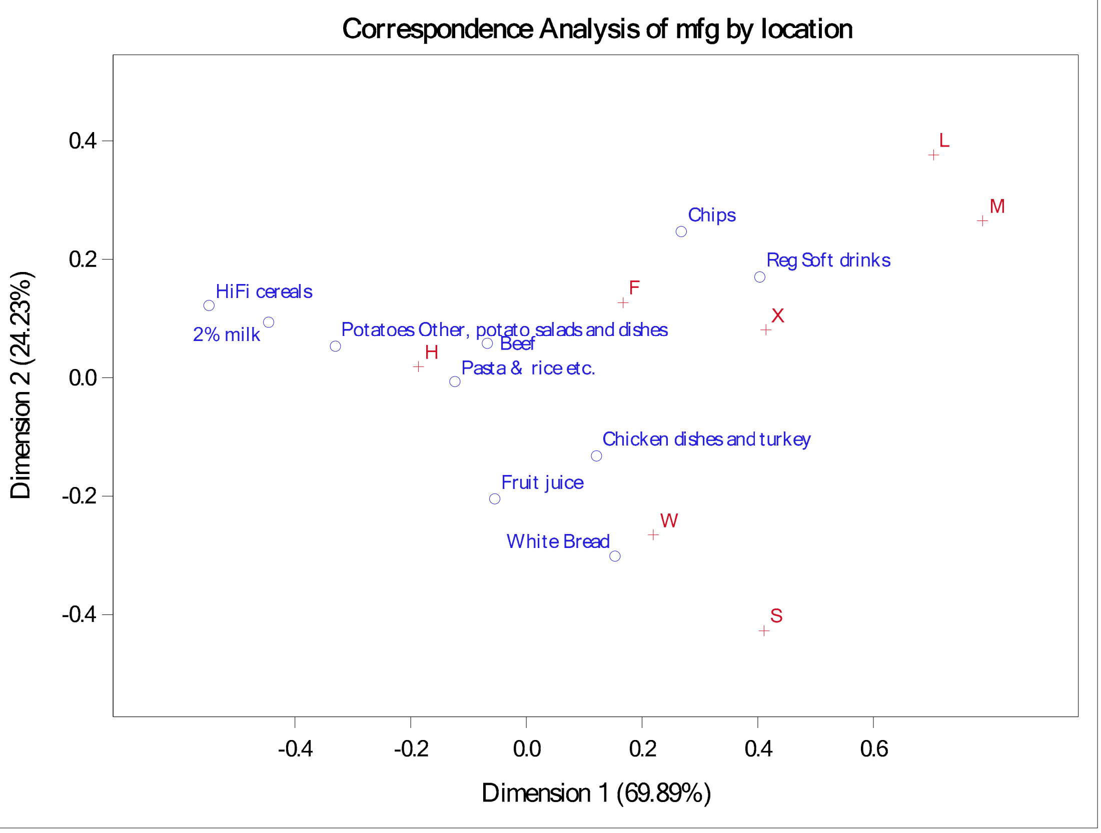

Figure S6: Correspondence analysis plot for Neutral Foods, with adjusted NDNS weights (individual's weight divided by number of food entry for that individual)

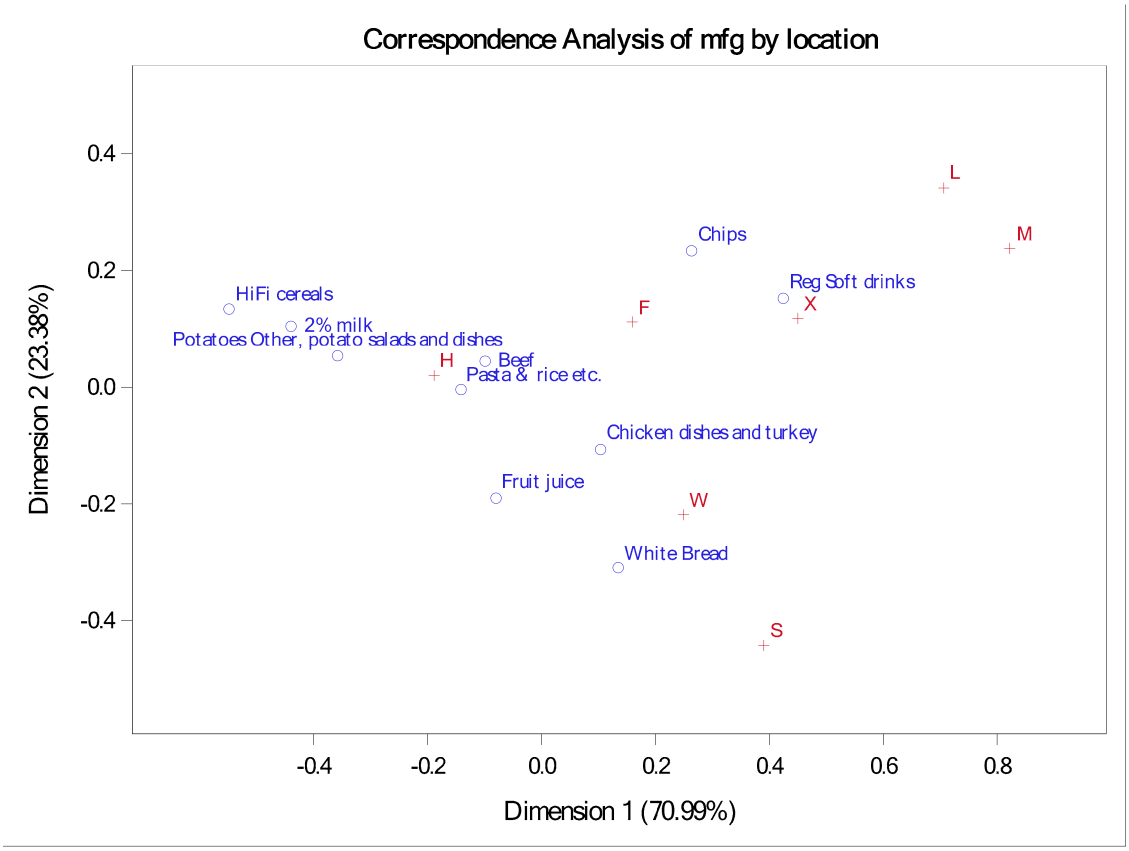

Figure S7: Correspondence analysis plot for Less-Healthy Foods, without NDNS weights.

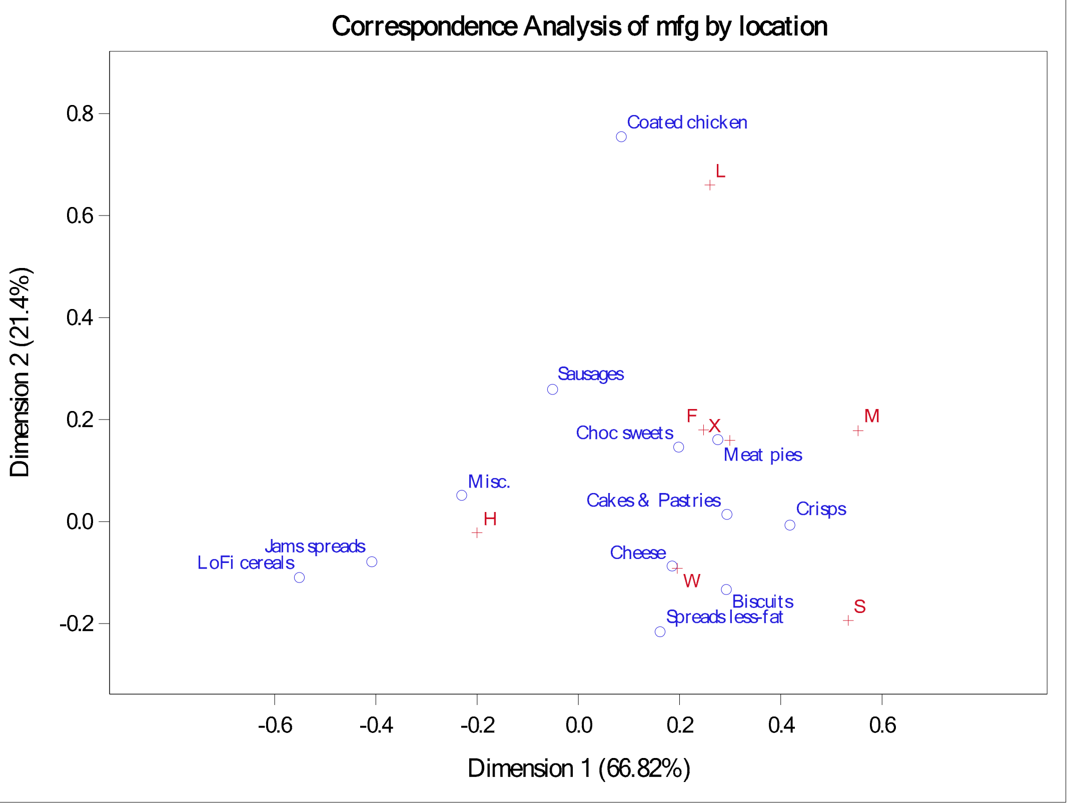

Figure S8: Correspondence analysis plot for Less-Healthy Foods, with individual NDNS weights.

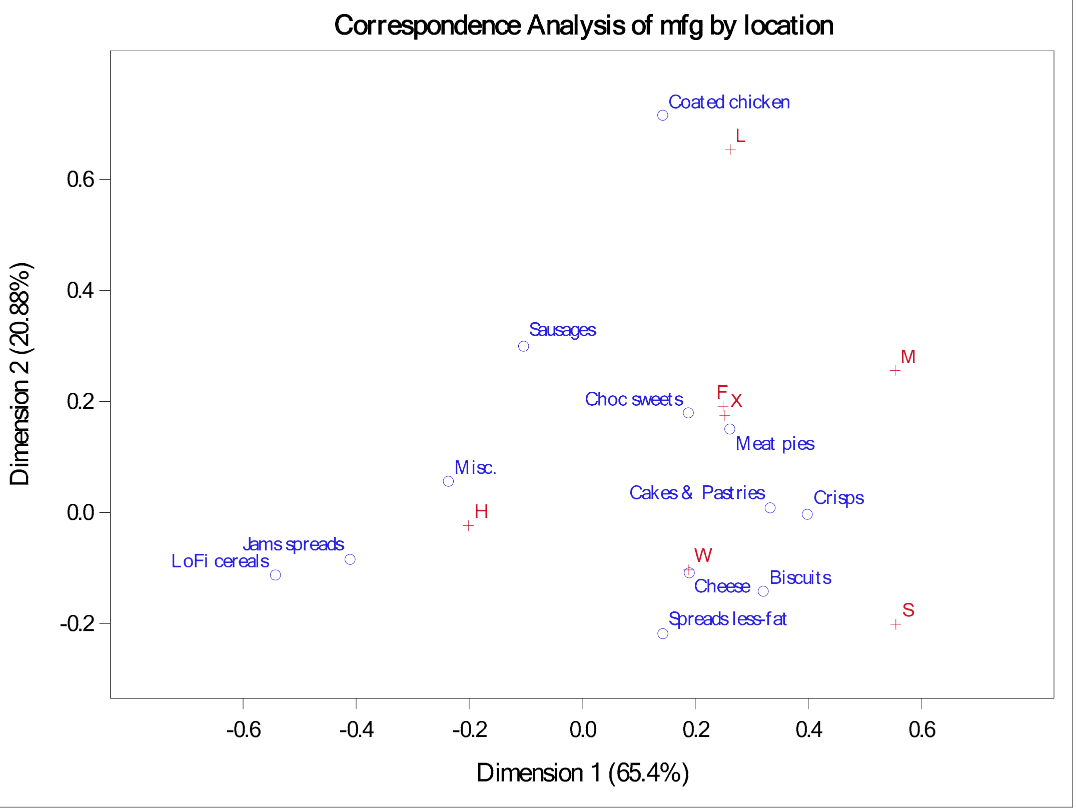

Figure S9: Correspondence analysis plot for Less-Healthy Foods, with adjusted NDNS weights (individual's weight divided by number of food entry for that individual)

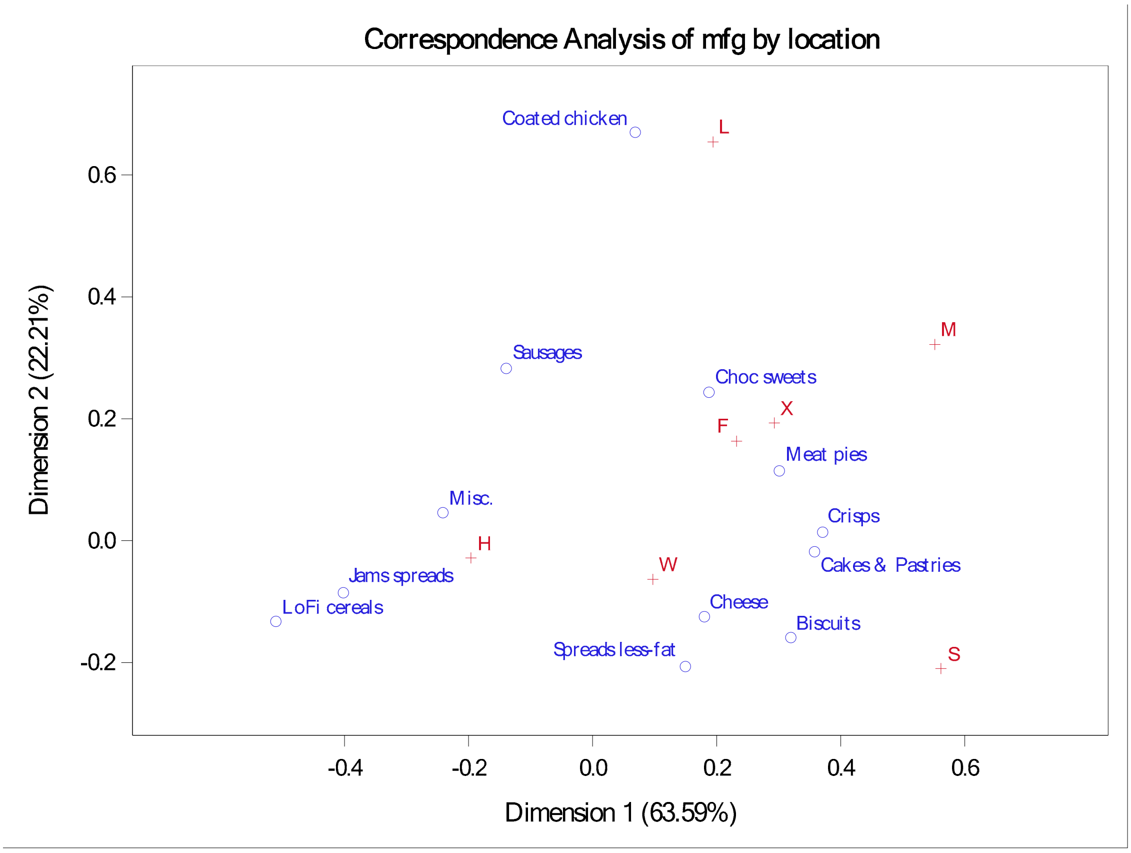

Figure S10: Correspondence analysis including all 25 main (P80) food groups for the 2008-2012 survey sample.

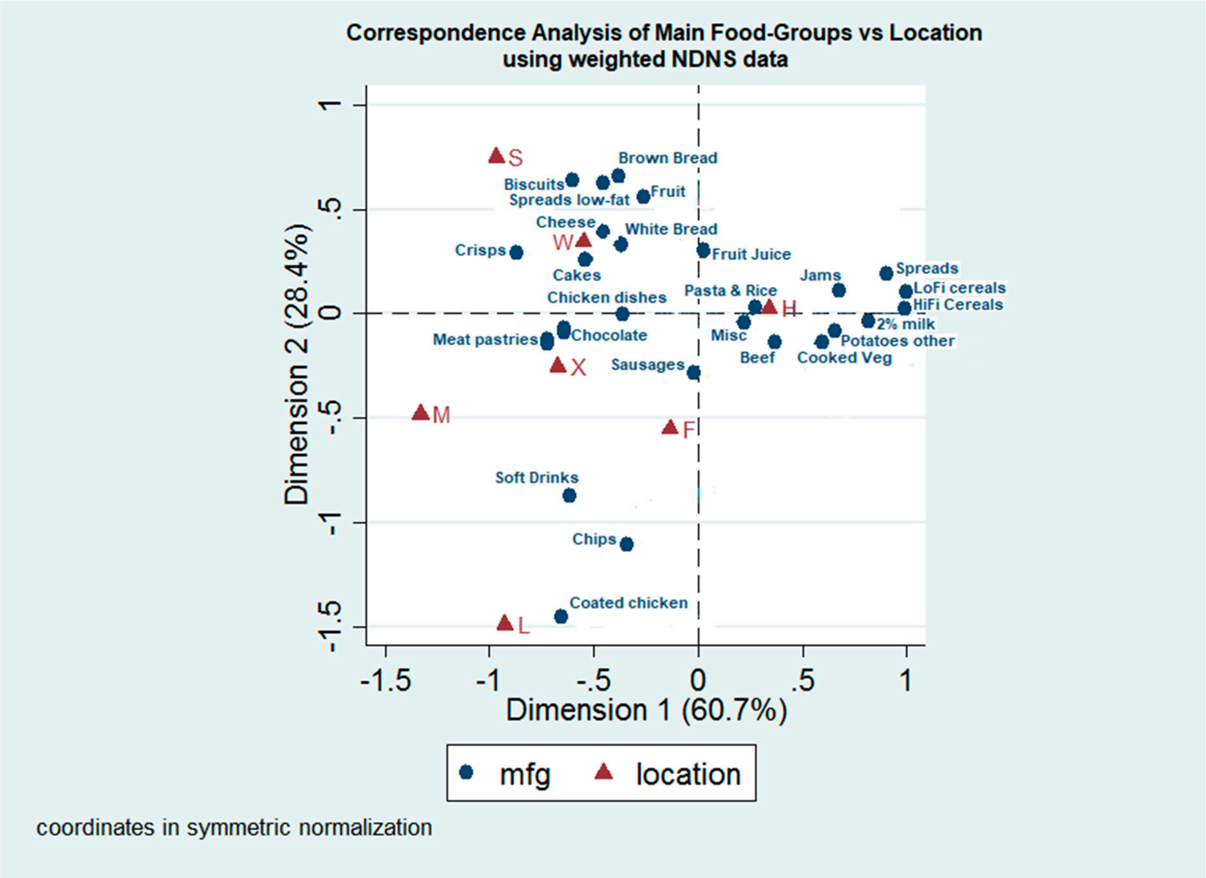

Supplement: Supplementary file 1 [file nutrients-12-02235-s001.pdf]
